# Translational studies of goal-directed action as a framework for classifying deficits across psychiatric disorders

**DOI:** 10.3389/fnsys.2014.00101

**Published:** 2014-05-26

**Authors:** Kristi R. Griffiths, Richard W. Morris, Bernard W. Balleine

**Affiliations:** Behavioural Neuroscience Laboratory, Brain and Mind Research Institute, University of SydneyCamperdown, Sydney, NSW, Australia

**Keywords:** goal-directed action, basal ganglia, amygdala, schizophrenia, ADHD, depression

## Abstract

The ability to learn contingencies between actions and outcomes in a dynamic environment is critical for flexible, adaptive behavior. Goal-directed actions adapt to changes in action-outcome contingencies as well as to changes in the reward-value of the outcome. When networks involved in reward processing and contingency learning are maladaptive, this fundamental ability can be lost, with detrimental consequences for decision-making. Impaired decision-making is a core feature in a number of psychiatric disorders, ranging from depression to schizophrenia. The argument can be developed, therefore, that seemingly disparate symptoms across psychiatric disorders can be explained by dysfunction within common decision-making circuitry. From this perspective, gaining a better understanding of the neural processes involved in goal-directed action, will allow a comparison of deficits observed across traditional diagnostic boundaries within a unified theoretical framework. This review describes the key processes and neural circuits involved in goal-directed decision-making using evidence from animal studies and human neuroimaging. Select studies are discussed to outline what we currently know about causal judgments regarding actions and their consequences, action-related reward evaluation, and, most importantly, how these processes are integrated in goal-directed learning and performance. Finally, we look at how adaptive decision-making is impaired across a range of psychiatric disorders and how deepening our understanding of this circuitry may offer insights into phenotypes and more targeted interventions.

## Goal-directed action and its relevance to psychiatry

Flexible behavior is fundamental for adapting to a changing environment. In this context, learning the consequences of an action and the value of those consequences are critical precursors for choosing the best course of action. Impairment in either process, or a failure to integrate them with action selection, leads to aberrant decision-making, with detrimental consequences for achieving goals and real-world functioning. Dysfunctional decision-making is common across a range of psychiatric disorders, and indeed, it has been argued that many psychiatric symptoms are associated with dysfunction in either learning or reward circuitry (cf. Nestler and Carlezon, 2006; Martin-Soelch et al., 2007). Determining how the brain supports each step in achieving flexible, goal-directed behavior is, therefore, not only a major goal of decision neuroscience, but may also provide valuable insight into the neurobiology and attendant functional disabilities associated with psychiatric illness.

Decades of research in associative learning have provided key insights into the behavioral and biological processes that mediate goal-directed action. One advantage of this approach has been the development of testable structural and functional hypotheses, and the invention of critical behavioral paradigms specifically to assess predictions from these hypotheses. We argue that this approach provides a unique opportunity to systemically explore the decision-making deficits commonly observed in clinical populations, and allows for the classification of a variety of decision-making impairments within a common framework. In this review, we first describe the psychological determinants of goal-directed behavior, and the evidence for how these processes map onto specific neural circuits. We will then use this framework to assess how these processes may be affected in common symptoms within three clinical disorders: schizophrenia, attention-deficit hyperactivity disorder (ADHD), and depression. Behavioral and neurobiological heterogeneities exist within traditional disorder classifications, as well as commonalities across diagnostic boundaries. We argue that knowledge of specific decision-making processes and their neural bases may provide a unifying framework, using which we can classify deficits across psychiatric disorders to produce a functionally–and biologically -driven understanding of psychopathology.

## What is goal-directed action?

Formally, goal-directed action reflects the integration of two sources of information: (1) knowledge of the causal consequences or outcome of an action; and (2) the value of the outcome (Dickinson and Balleine, 1994; Balleine and Dickinson, 1998). The integration of both of these features, causal knowledge and reward value, is essential in producing goal-directed actions. Impairments in such actions can arise through a deficiency in either process, or through an inability to integrate them appropriately to guide decision-making. We will first discuss each of these features in turn and the key neural substrates that current research suggests are involved in these processes. We will then turn to potential deficits in these processes using examples of specific psychopathology, and in particular, how they are related to symptoms common to depression, schizophrenia and ADHD.

### Causal learning and action-outcome encoding

Knowledge regarding the causal consequences of specific actions emerges from the experienced contingency. Such contingencies can be positive, promoting performance of an action, or inhibitory; i.e., in some situations actions may prevent a desired outcome and, in these situation, actions should be withheld (Dickinson, 1994). Considerable research using tasks such as the Iowa Gambling Task (IGT; Bechara et al., 1994) and the Wisconsin Card Sorting Task (WCST; Grant and Berg, 1948) suggests that humans and rats are exquisitely sensitive to feedback contingent on their actions, and can flexibly update their choices based on that feedback. However, because specific choice problems are signaled using unique discriminative or localized cues in these tasks, choice performance could reflect knowledge of the action-outcome contingency or associations between the action or the outcome with these task-related cues. This is a non-trivial distinction; as we shall review below, research has shown that different psychological processes and neural circuits exert control when actions are guided by environmental stimuli or by the action-outcome contingency (see Balleine and Ostlund, 2007; Balleine and O’Doherty, 2010 for reviews).

Experimentally, we are able to determine the degree to which choice is guided by the action-outcome contingency using contingency degradation tests. In such tests a specific action-outcome contingency is degraded by introducing an outcome in the absence of its associated action, thereby reducing the causal relationship between them. This treatment decreases the performance of the degraded action in goal-directed agents (Hammond, 1980; Balleine and Dickinson, 1998). For example, Balleine and Dickinson trained rats to perform two actions, lever pressing and chain pulling, with one action earning sucrose and the other, food pellets. They subsequently delivered one of the two outcomes non-contingently, such that the probability of receiving that outcome was the same whether the rat performed its associated action or not. This produced a selective decrease in the performance of the degraded action. Similarly, it has been demonstrated in healthy humans that the degree of contingency degradation is negatively correlated with the rate of performance and with judgments regarding how causal an action is with respect to its outcome (Shanks and Dickinson, 1991; Liljeholm et al., 2011).

### A specific corticostriatal circuit mediates the causal effects of actions

**Figure 1:**
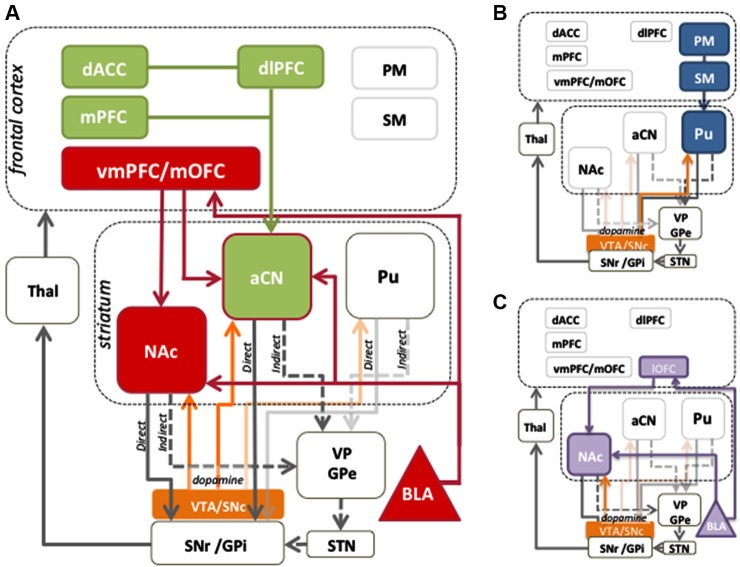
**Cortico-striatal circuits involved in instrumental conditioning.** **(A)** Evaluative learning processes, shown in red, are mediated by bilateral connections between the medial orbitofrontal cortex (mOFC) and basolateral amygdala (BLA), which are relayed to the anterior caudate nucleus (aCN). Contingency learning processes, shown in green, are thought to occur in the medial prefrontal cortex (mPFC) and are relayed to the aCN to mediate control of action selection. Reward information is also relayed to the nucleus accumbens (NAc) to provide motivational drive for the performance of instrumental behaviors. The dlPFC and dorsal anterior cingulate cortex (dACC) play a role in comparing action values and can exert a modulatory influence over circuits involving prefrontal and aCN activity. Together, the contingency and evaluative circuits allow for the acquisition of goal-directed behaviors. **(B)** Stimulus-response associations, or habits, are mediated by projections from premotor (PM) and sensorimotor cortices (SM) to the posterior putamen (Pu). **(C)** The lateral orbitofrontal cortex (lOFC) and the BLA encode the value assigned to reward predictive stimuli, which the NAc uses to mediate instrumental performance. Mid-brain dopamine modulates plasticity in the dorsal striatum, and is associated with motivational processes in the ventral striatum. The balance between striatal output to the direct (D1) and indirect (D2) pathways serves to promote or inhibit behavior, respectively.

Systematic use of contingency degradation tasks in rodent studies has identified specific regions of prefrontal cortex and dorsomedial striatum necessary for encoding the action-outcome contingency (Corbit and Balleine, 2003; Yin et al., 2005; Lex and Hauber, 2010). In humans, there is evidence that homologous regions to those in rodents, i.e., the medial prefrontal cortex (mPFC) and anterior caudate, play a similar role in contingency sensitivity (cf. Balleine and O’Doherty, 2010). Tanaka et al. (2008) and Liljeholm et al. (2011) manipulated experienced action-outcome contingencies, and observed positive modulation of blood oxygenation level dependent (BOLD) activity in the human mPFC, and anterior caudate nucleus (aCN). Furthermore, mPFC activity reflected the local experienced correlation between responding and reward delivery, consistent with a role in the online computation of contingency (Tanaka et al., 2008). Activation of the aCN can also occur even fictively, in cases where a contingency between action and outcome is perceived where one does not actually exist (Tricomi et al., 2004), whereas subjective causality judgments have been shown to correlate with activity in the mPFC, along with the dorsolateral prefrontal cortex (dlPFC), a region implicated in top-down cognitive control (Tanaka et al., 2008). As shown in the green in Figure 1A, these data suggest that signals produced in the mPFC may be relayed to the aCN, where changes in contingency can be assimilated with evaluative information from other cortical regions.

Further evidence for the importance of the caudate in contingency sensitivity and in guiding action selection comes from studies in non-human primates. Samejima et al. (2005) recorded from striatal neurons during a choice task in which monkeys made left or right actions to obtain reward. Importantly, on some trials, action-outcome contingencies were similar whereas on others they differed so that activity related to the action value (in this instance, the strength of the action-outcome contingency) could be dissociated from the motor choice. They found that a large number of striatal neurons encoded action values, which subsequently influenced the probability of selecting a particular action. Lau and Glimcher (2007) also found populations of neurons in the caudate that encoded actions and outcome post-choice. The temporal correlation of neuronal firing rates with behavior suggested that the caudate not only represents the contingency of potential options, but might also update this information once the outcome has been received.

### The role of value in goal-directed decision-making

In addition to causal knowledge, determining the current value of available outcomes in the context of current internal states or contexts is also critical for adaptive decision-making. For example, a state of hunger increases the desirability or incentive value of food relative to a satiated state, and increases its motivational impact. Outcome revaluation procedures exploit these variations in value. A common means of changing the value of a specific food is using sensory-specific satiety (Rolls et al., 1981). For example, in studies in which rats were trained to perform two actions for distinct outcomes, giving them an extended opportunity to eat one or other outcome altered the desirability of that outcome without affecting the value of the other uneaten outcome (Balleine and Dickinson, 1998). When given the opportunity to choose between the two actions in the absence of any reward delivery (to prevent learning about the association between the action and the new outcome value during the test) the rats clearly preferred the action that had previously earned the outcome they had not eaten. Selective decreases in the performance of actions associated with a devalued outcome provide clear evidence that, in conjunction with knowledge of the action-outcome contingency, action selection is governed by the current value of the outcome.

An alternate means of revaluing the outcome used in animal research is conditioned taste aversion whereby an outcome is paired with a mild toxin such as lithium chloride that induces gastric malaise. In humans disgust can also be a useful tool for devaluing outcomes. For instance, food desirability ratings can be decreased considerably when an otherwise preferred outcome has been paired with an aversive taste (e.g., Baeyens et al., 1990).

### The OFC and vmPFC play a role in encoding value relative to the current motivational state

The OFC and, more broadly, the vmPFC, illustrated in red in Figure 1A, have long been argued to be critical for signaling the current value of an outcome. Single unit recording studies in hungry non-human primates found unit responses in the caudolateral OFC during presentation of a pleasant odor or taste, which decreased to baseline when the monkey were satiated (Rolls et al., 1989). Similarly, when humans were presented with food outcomes, the degree of hunger and pleasantness caused graded OFC/vmPFC BOLD activity (Morris and Dolan, 2001; Kringelbach et al., 2003) that was reduced after satiation with the presented food (O’doherty et al., 2000; Small et al., 2001; Valentin et al., 2007). Interestingly, this reduction in activity was evident even when using instructed devaluation, where participants were simply told via a red X over a predictive stimulus that the outcome was no longer valuable (de Wit et al., 2009) suggesting that revaluation, whether through visceral or cognitive treatments, affects value via a common neural pathway. These data advance the idea that the OFC undertakes simple economic valuation and emphasize its role in determining outcome value in the context of the *current* motivational state. Jones et al. (2012) have further developed this idea, arguing that the OFC is required when value is inferred from associative structures (i.e., value is computed based on the current state), but not when relying on pre-computed values stored from previous experience.

It is important to note that BOLD activation during evaluation has been reported within both the lateral and medial portions of the OFC. There is, however, evidence for cytoarchitectural and functional heterogeneity within the OFC (Carmichael and Price, 1995; Elliott et al., 2000; Kahnt et al., 2012), suggesting that studies using reward-predictive cues are utilizing alternate or additional learning processes. Though there is still considerable debate on this topic, a converging view is that the mOFC is involved in updating the expected values of different experienced outcomes, whereas the lateral OFC is responsible for the formation and updating of values derived from Pavlovian stimulus-outcome associations (Walton et al., 2010; cf Balleine et al., 2011; Fellows, 2011; Noonan et al., 2011, 2012; Rudebeck and Murray, 2011; Klein-Flügge et al., 2013). Both the predicted value of an outcome based on the presence of a Pavlovian cue, and the experienced value of an instrumental outcome, are incentive processes that play an important role in motivating behavior. Due to the differing circuitry and learning processes (instrumental vs. Pavlovian) however, paradigms that disentangle these processes provide clearer information.

### The influence of a limbic cortico-striatal circuit on the value of outcomes and cues that predict outcome delivery

Whereas the mOFC is computing current outcome value, the basolateral amygdala (BLA) plays a more fundamental role, linking value information with the sensory features of the reward or reward-related cues (see Figure 1A). A series of studies by Balleine et al. (2003) found that lesions of the BLA attenuated the sensitivity of rats to outcome devaluation, both when tested in extinction and with the outcome present. Furthermore, BLA lesions have been found to abolish the selective excitatory effects of reward-related cues whilst sparing the general motivational effects that such cues exert over responding (Corbit and Balleine, 2005). In humans, Jenison et al. (2011) acquired single neuron recordings from the BLA whilst subjects made monetary bids on food items that were presented to them as pictorial stimuli. Firing rates were linearly related to the monetary value assigned to food item stimuli, supporting a role for the BLA in assigning value to stimulus events. The strength of association between incentive value (either positive or negative) and both the features of outcomes and predictive cues not only determines their valence but also the magnitude of evaluative judgments, in keeping with a range of human imaging studies that have concluded the amygdala provides an overall magnitude signal for value judgments, or the interaction between intensity and valence (Anderson et al., 2003; Arana et al., 2003; Small et al., 2003; Winston et al., 2005).

Extensive anatomical connectivity exists between the OFC and BLA (see Figure 1A; Stefanacci and Amaral, 2002; Ghashghaei et al., 2007) allowing them to work closely together in encoding and retrieving value information (see Holland and Gallagher, 2004, for a review). Indeed, damage to the BLA can produce similar deficits to those observed from damage to the OFC (Hatfield et al., 1996; Baxter et al., 2000). However, no brain region acts in isolation, something clearly demonstrated when brain structures are left intact and only their anatomical connections with other structures are severed. Using OFC-BLA contralateral disconnection lesions, Zeeb and Winstanley (2013) found that rats were unable to update their choice preference following reward devaluation. This effect occurred both when the reward was delivered during test and also during extinction when rats needed to rely on stored representations of the outcome. The rats with disconnected OFC and BLA, however, did not differ from controls in their press rates or response latencies, suggesting an impairment specific to altering the value of a particular reward rather than a general reduction in motivation. Similar effects have been observed in humans where structural and functional connectivity between the OFC and BLA was found to correlate with rate of acquisition on a reversal learning task (Cohen et al., 2008).

The nucleus accumbens (NAc) also receives excitatory afferents from the OFC and BLA (amongst other regions), and selectively gates information projecting to basal ganglia output nuclei (Figure 1A; Alheid and Heimer, 1988; Groenewegen et al., 1999). It is often described as the limbic-motor interface, mediating the effect of reward value on action selection (Mogenson and Yim, 1991). Lesions of the NAc core impair the ability of rats to selectively reduce responding after outcome devaluation, demonstrating reduced sensitivity of instrumental performance to changes in outcome value (Corbit et al., 2001; Corbit and Balleine, 2011; Laurent et al., 2012) Importantly, lesions of the NAc also cause a reduction in the vigor of performance, indicating that this region may be involved in how the general motivating properties of reward-related stimuli affect performance (Balleine and Killcross, 1994; Corbit et al., 2001). Interestingly, NAc lesions do not impair sensitivity to selective contingency degradation, revealing that this region does not itself encode the action-outcome contingency but, rather, brings changes in reward value to bear on performance (Corbit et al., 2001). These key evaluative circuits are represented by the red connections in Figure 1A.

### Action values: the integration of contingency and value

The value of an action is a product of its contingency with a particular outcome and the desirability of that outcome. As a consequence, interest has grown in the analysis of the neural circuits involved in computing these action values. Studies using trial-and-error action-based learning tasks have reported action value-related signals in the supplementary motor area, where actions are presumably planned before execution. In contrast, BOLD activity in the vmPFC was modulated by the expected reward signal of the chosen action, suggesting that this region provides the agent with feedback about the consequences of their actions to guide future choices (Gläscher et al., 2009; Wunderlich et al., 2009; FitzGerald et al., 2012; Hunt et al., 2013). Camille et al. (2011) found that humans with dorsal anterior cingulate cortex (dACC) damage were unable to maintain the correct choice between actions after positive feedback, suggesting that this region is critically involved in updating action values, perhaps passing feedback from the vmPFC to the action planning areas in the supplementary motor areas via the aCN.

Top-down cognitive control exerted by such structures as the dlPFC and dACC may also modulate the integration of value and contingency, and its conversion into performance. Kim and Shadlen (1999) and Wallis and Miller (2003) found dlPFC neurons that encoded both reward value and the forthcoming response, whereas Kim et al. (2008) found neurons that ramped up or down in their firing rate with increasing or decreasing action values until a choice was made. In the ACC, neural signals resembling the difference between action values, or a combination of movement intention and reward expectation, have been reported (Matsumoto et al., 2007; Seo and Lee, 2007; Wunderlich et al., 2009). Furthermore, lesions of this area in non-human primates and humans produces deficits in action-based choice (Kennerley et al., 2006; Camille et al., 2011). Although there is less agreement about the distinctions in function of the dlPFC and ACC, it is clear that disturbances within these regions radically alter goal-directed choice.

We do know however that the anterior caudate, a part of the associative striatum, is a critical node in the goal-directed network, receiving evaluative input from the BLA and OFC, as well as contingency input from the dlPFC and mPFC. This is supported by data showing that the integration of dopamine and glutamate neurotransmission within this region enables learning and action control by shaping synaptic plasticity and cellular excitability (Shiflett and Balleine, 2011a). In particular, the extracellular signal-regulated kinase (ERK) is particularly important for goal-directed action control due to its sensitivity to combined DA and glutamate receptor activation (Shiflett et al., 2010; Shiflett and Balleine, 2011b). Thus, perturbation of ERK activation associated with various forms of psychopathology and/or drug abuse may produce deficits in goal-directed control. Nevertheless, the role of this region in mediating information from limbic and cortical networks has only relatively recently been recognized in other forms of psychopathology such as that involved in schizophrenia (Howes et al., 2009; Kegeles et al., 2010; Simpson et al., 2010).

### Summary of neurobiology of goal-directed learning

In summary, the vmPFC is a functionally complex region critically involved in networks that compute and update outcome values based on feedback or changes in state. The BLA assists in this process by associating incentive value with the sensory information that informs the agent of the reward properties of outcomes, whilst the NAc brings this evaluative information to bear on performance. Simultaneously, the associative striatum and mPFC are also involved in the learning of action-outcome associations, providing information on how to obtain desired outcomes. Together, these processes are integrated in the associative striatum to produce goal-directed behavior. For the purpose of brevity, we have focused on what we believe are the key neural regions involved in goal-directed learning. It must be acknowledged, however, that many other regions likely contribute to these processes in ways that are not yet fully understood.

## Stimulus-driven effects on instrumental behavior

Multiple learning systems are involved in the production of healthy everyday behavior. So far we have focused on behavior guided by goals rather than cues. Goal-directed processes allow for flexible choices in the face of changing environmental contexts and conditions. Under stable conditions however, the consequences of actions need not be continually assessed. In these instances, habitual actions, established by the formation of stimulus-response associations, allow reflexive, cue-driven responses to occur at higher speeds and with lower cognitive load (see Figure 1B). The associative systems mediating goal-directed actions and habits are thought to coexist and compete for behavioral control in adaptive decision-making (Dickinson and Balleine, 1993). Another major learning process influencing behavior is the formation of Pavlovian stimulus-outcome associations and conditioned responding (see Figure 1C). Cues associated with reward are able to evoke reward anticipation, which may subsequently guide or bias instrumental choices. Both reward-predictive cues and the experienced value of an instrumental outcome are important incentive processes that play an essential role in motivated behavior. Importantly however, although both may be able to induce reward approach behavior, Pavlovian cues exert their effects on actions through stimulus, rather than outcome value, control.

As depicted in Figure 1, these learning systems are situated in functionally organized cortico-basal ganglia loops. The cortical regions of each system send topographically organized inputs to the striatum—motivational or limbic input to the ventral striatum, associative input to the aCN and anterior putamen, and sensorimotor input to the posterior putamen (Nakano, 2000). From the striatum, GABA-ergic medium spiny neurons (MSNs) project to the principle striatal output nuclei, the substantia nigra pars reticulata (SNr) either directly or indirectly via the globus pallidus pars externa (GPe) and subthalamic nucleus (STN). Whereas MSNs in the direct pathway predominantly express dopamine D1 receptors and activate behavioral functions, those in the indirect pathway express dopamine D2 receptors and tend to suppress behavior (Albin et al., 1989). The ascending dopaminergic system, projecting to the striatum from the substantia nigra pars compacta (SNc) and ventral tegmental area (VTA), plays an important role in modulating activity within these pathways due to their differential expression of D1 and D2 receptors. These modulate the activity of the MSNs bidirectionally; whereas dopamine increases the activity in D1 expressing MSNs, it reduces the activity of D2 expressing MSNs (Gerfen and Surmeier, 2011).

## The breakdown of goal-directed processes in psychiatric and neurodevelopmental disorders

The nature of the interaction and cooperation between goal-directed and habitual control processes during decision-making has particular implications should problems arise in the cognitively demanding goal-directed system. Under such conditions, behavioral control may become dominated by dysregulated habitual control, resulting in the loss of flexibility of thought, and the increased stereotypy and behavioral disinhibition characteristic of many psychiatric conditions. Deficits in incentive processes may also produce a range of motivational dysfunctions. Having outlined these processes and their interaction in healthy decision-makers, together with the key neural systems involved above, we turn to consider whether deficits in goal-directed decision-making in psychiatric disorders map onto a common framework. Here we review select evidence for patterns of deficits in outcome sensitivity, action-outcome contingency awareness, and in the integration of these features with action selection in three disorders known for their motivational and cognitive deficits: schizophrenia, ADHD and depression.

### Schizophrenia

Motivational and associative learning dysfunction have long been noted in schizophrenia, and have been implicated in positive, negative and cognitive symptomology (Gold et al., 2008). It is often noted that individuals with schizophrenia experience difficulties using emotional states, prior rewards and goals to drive goal-directed action (Barch and Dowd, 2010); i.e, the relationship between value representations and action selection appears to be lost (Heerey and Gold, 2007; Gold et al., 2008; Heerey et al., 2008). We propose that this is due to what amount to functional disconnections within the cortico-striatal loops responsible for integrating evaluative and contingency learning for goal-directed action selection.

#### Reduced sensitivity to changes in reward value

Negative symptoms such as anhedonia (an inability to experience pleasure) and avolition (a reduced motivation to engage in motivated goal-directed behavior) seem to suggest valuation and action selection deficits are primary in this disease. Anhedonia may be produced by a breakdown in the evaluative circuits responsible for the actual consummatory pleasure experienced from the reward (i.e., the red circuit in Figure 1A). Recently however, a number of studies have shown that, on experiencing or consuming rewards, hedonic ratings are often not significantly reduced compared to controls (Burbridge and Barch, 2007; Gard et al., 2007; Heerey and Gold, 2007) and we have found similar effects in the lab. If evaluative learning is intact, then the critical deficit may lie in anticipating hedonic consequences (reward value) or in using experienced reward values to guide action-selection. Numerous behavioral and neuroimaging studies have focused on whether patients can anticipate reward values. For example, patients with severe avolition fail to choose stimuli associated with monetary reward over a stimulus indicating the avoidance of monetary loss (i.e., no reward) (Gold et al., 2012). This deficit in reward anticipation is consistent with neuroimaging evidence that ventral striatal responses to cues predicting reward are dulled in schizophrenia (Juckel et al., 2006a), including amongst unmedicated patients (Juckel et al., 2006b). Patients also have aberrant neural responses to rewards themselves, including predicted and unpredicted rewards (Waltz et al., 2009; Morris et al., 2012). However no study to date has tested whether patients can adjust their actions solely on the basis of experienced reward values. In a recent study, we tested whether patients with schizophrenia could use the anticipated or experienced reward value to select actions. Patients were able to learn action-outcome associations, and subjectively reported reductions in outcome value after an outcome devaluation procedure, however they did not use this updated outcome knowledge to effectively guide their choices, suggesting that the ability of patients to integrate the values of rewards with action selection processes is deficient. Importantly, BOLD activity in the caudate nucleus during the test requiring this integration was also deficient in patients. Moreover, reduced neural responses in the head of the caudate predicted more severe negative symptoms. This is consistent with recent evidence that neuropathology in schizophrenia, including upregulation of striatal D2 receptor density and occupancy, is most prevalent in the associative regions of the striatum (Buchsbaum and Hazlett, 1998; Abi-Dargham et al., 2000; Howes et al., 2009; Kegeles et al., 2010). On the other hand, patients were able to select actions on the basis of the anticipated reward value, when a cue predicting the availability of reward was presented, albeit not to the same extent as healthy adults (Balleine and Morris, 2013). Thus, the integration of reward values with action selection appears to be impaired in schizophrenia. This particularly affects goal-directed actions when cues are not present to indicate the consequences of action.

The caudate is a critical site for goal-directed actions but it does not function in isolation. In addition to aberrant regional activity in schizophrenia, there is also evidence for functional disconnection of the caudate from its cortical afferents, which can also be found during the prodromal state (Buchsbaum et al., 2006; Yan et al., 2012; Fornito et al., 2013; Quan et al., 2013; Quidé et al., 2013; Wadehra et al., 2013). Thus, the caudate-cortical disconnection in schizophrenia is a critical target for understanding the deficit in goal-directed behavior and predicting functional outcomes associated with the disease.

#### Changes in contingency awareness

Cognitive deficits are the most pervasive and difficult to treat aspects of schizophrenia (Green, 1996). In particular, any deficit in the ability to form and use A-O associations appropriately and learn about the consequences of our everyday choices is likely to have a large impact on social and occupational functioning. Multiple studies have suggested that the initial acquisition of probabilistic contingencies is relatively unimpaired in schizophrenia, with the exception of some reports of slower rates of acquisition (Weickert et al., 2002; Kéri et al., 2005; Waltz and Gold, 2007). When contingencies are reversed many studies have shown schizophrenic patients do show significant impairments (Waltz and Gold, 2007; Murray et al., 2008), suggesting patients are insensitive to changes in action-outcome contingency. However, distinguishing this impairment in reversal learning from slower acquisition more generally has not been convincingly demonstrated. Using cognitive modeling, however, Strauss et al. (2011) found that patients with schizophrenia have a reduced tendency to explore alternative actions in an uncertain environment. This perseverative style of responding during uncertainty is consistent with greater habitual control of actions. A weakened sensitivity to the action-reward correlation and the predominant use of an S-R learning strategy is also consistent with the fact that rapid learning from trial-by-trial feedback is often impaired but more gradual learning remains intact (Kéri et al., 2005; Gold et al., 2008).

At a neural level, the associative striatum plays an integral role in acquiring A-O contingencies, detecting contingency changes and flexibly using this information during the process of action selection. As reviewed above, functional deficits in the associative striatum as well as pathology in cortical afferents appear early in the pathogenesis of schizophrenia and may be a risk factor for the disease. In this case, a deficit in learning action-outcome contingencies, which critically depends on this circuit, may stand in as an important marker of brain function. However, at present the status of contingency learning deficits in schizophrenia is unclear. Reversal learning tasks such as the IGT or the WCST are generally controlled by reward-related stimuli rather than by the relationship between action and outcome, which makes it difficult to discern whether any deficits are due to altered Pavlovian or instrumental learning. In addition, in reversal learning tasks, it is difficult to establish whether changes in outcome value or in contingency are driving choices. Thus, the use of contingency degradation tasks within this cohort will be critical to provide convincing evidence regarding the level of impairment in contingency awareness and the functional status of the related circuits.

#### Schizophrenia summary

In summary, during goal-directed learning, patients with schizophrenia are only mildly or are unimpaired in their subjective valuation assessments, and in the activation of prefrontal regions that support them. Dysfunction in the associative striatum and its cortical afferents, however, may interfere with the ability to modulate action selection using value information. Evidence also suggests that patients with schizophrenia are able to encode initial A-O associations, but they may be impaired at updating associations for flexible use in action selection. Taken together, these impairments in integrating the key components of goal-directed behavior suggest that patients with schizophrenia may over rely on habit learning and habitual strategies, predicting relatively intact functioning of the circuitry mediating habitual control but not goal-directed performance.

### ADHD

Altered sensitivity to reinforcement is acknowledged as an important etiological factor in a number of theoretical frameworks of ADHD (Barkley, 1997; Sergeant et al., 1999; Castellanos and Tannock, 2002; Sagvolden et al., 2005; Frank et al., 2007; Tripp and Wickens, 2008; Sonuga-Barke and Fairchild, 2012). ADHD is characterized by symptoms of inattention, hyperactivity and impulsivity, consistent with dysregulation of top-down control processes modulating goal-directed control. A number of researchers have argued that ADHD is a motivational problem, whereby individuals are unable to use intrinsic motivation to guide choice performance (Douglas, 1989; Sergeant et al., 1999). This is supported by evidence that children with ADHD perform well on continuous reinforcement schedules, whereas their performance deteriorates on partial reinforcement schedules where the consistent extrinsic motivation of reward is not provided (Parry and Douglas, 1983; Luman et al., 2008).

Dopaminergic dysfunction clearly plays a key role in ADHD symptomology. The primary treatment for ADHD, Methylphenidate, preferentially blocks the reuptake of DA in the striatum (Schiffer et al., 2006), and studies have demonstrated its effectiveness in normalizing reinforcement sensitivity in ADHD relative to placebo (Tripp and Alsop, 1999; Frank and Claus, 2006). Furthermore, Volkow et al. (2012) has proposed that disruption of D2/D3 receptors is associated with the motivation deficits observed in ADHD, which may in turn contribute to attention deficits. Attention was found to be negatively correlated with D2/D3 receptor availability in the left NAc and caudate (Volkow et al., 2009), regions key to reward valuation and contingency awareness in goal-directed action. We hypothesize that motivational problems stem primarily from an inability to predict the rewarding consequences of cues or actions. As a consequence actions may be poorly controlled or regulated resulting in inappropriate responses to the situation and undesirable consequences.

#### The dopamine transfer deficit theory

The Dopamine Transfer Deficit theory of ADHD (Tripp and Wickens, 2008, 2009) proposes that altered phasic dopamine responses to reward-predictive cues results in blunted stimulus-outcome associations, and hence blunted reward anticipation. In this sense, motivational deficiencies may be derived from a lack of stimulus-outcome contingency awareness (i.e., an impairment within the circuitry detailed in Figure 1C). The relatively consistent finding of hypo-activation in the ventral striatum during reward anticipation supports this idea (Scheres et al., 2007; Ströhle et al., 2008; Plichta et al., 2009; Hoogman et al., 2011; Carmona et al., 2012; Edel et al., 2013; Plichta and Scheres, 2013). Wilbertz et al. (2012) found increased OFC activation during outcome delivery consistent with increased excitation to reward; however, as reward-related stimuli were generally less successful at inducing reward anticipation, it may also reflect an aberrant prediction error-like response. Overall, rather than suggesting that reward sensitivity is impaired, the evidence seems to support the notion that an inability to anticipate reward may reduce motivation or impair the ability to select the relevant action.

In comparison to schizophrenia, both patient groups have intact reward sensitivity, however the pathologies can be dissociated by the role of *predicted* reward-values and *experienced* reward-values on action-selection. In ADHD, we expect to see impairment in selecting actions on the basis of predicted reward (e.g., a deficit in outcome specific Pavlovian-to-instrumental transfer); whereas in schizophrenia the deficit is related to using experienced reward values to guide action selection (e.g., a deficit in outcome specific devaluation). The amount of overlap between these two groups should, therefore, be predicted to depend on the extent to which both share neuropathology in the ventral striatum, which will disrupt dopamine signaling due to hyper- or hypodopaminergia, regardless.

#### Incentive learning deficits, response inhibition and impulsivity

Response inhibition and impulsivity are key deficits exhibited in ADHD even when executive function demands are low (Wodka et al., 2007); both children and adult subjects are slower to inhibit responses during the go/no-go or stop-signal reaction time (SSRT) tasks, and make more errors than age-matched controls (Schachar et al., 1995; Purvis and Tannock, 2000; see Solanto, 2002 for a review). Lesions of the BLA and NAc both increase impulsive choice on a delay-discounting task in rats (Winstanley et al., 2004), and measures of impulsivity are generally negatively correlated with white matter integrity in right OFC fiber tracts in adults with ADHD. Thus impulsivity may be induced by dysfunction in key incentive processing regions, or alternatively, these regions may be underutilized due to an over reliance on reflexive actions that are not based on the value of consequences.

#### Changes in contingency awareness

Tripp and Wickens (2008) postulate that stimulus-outcome associations are disturbed in ADHD due to a lack of transfer of dopamine firing from reward receipt to reward-predictive cues. To date, however, there have not been any comparable studies assessing whether this is also the case for action-outcome learning. We predict that due to dopamine dysregulation within the associative striatum, contingency awareness will be deficient perhaps for both cue and action–based associations with specific outcomes. Firstly, reduced salience or attention allocation due to dysfunction in DA firing may inhibit the formation of action-outcome associations. Furthermore, when a temporal delay occurs between an action and its outcome, DA dysfunction may generate difficulties in “credit assignment”—deciding to which recent action one should attribute the outcome (Johansen et al., 2009). This difficulty could contribute to the delay aversion often documented in ADHD (Sonuga-Barke, 2002), and the easy distraction by extraneous stimuli. For instance, Carlson et al. (2000) found that, relative to controls, ADHD children were more likely to attribute success on an arithmetic task to luck, which seems to support reduced awareness of action-outcome causality. The dopamine transfer deficit theory also predicts that in ADHD, smaller anticipatory dopamine signals relative to the response to actual reinforcers would result in a greater influence of the most recent contingency than longer-term reinforcement history (Tripp and Wickens, 2008). This could result in faster extinction under partial reinforcement, or increases in the performance of occasionally rewarded, but overall suboptimal, actions.

#### Caudate impairments and action selection in ADHD

Meta-analyses have shown that the most consistent gray matter reductions in ADHD occur in the caudate, a region critical for goal-directed behavior. This morphological deficit was worse in samples with lower levels of stimulant medication, suggesting that dopamine normalization may counteract caudate atrophy (Valera et al., 2007; Nakao et al., 2011). Impairments in the striatum likely affect both contingency awareness and their integration with action selection processes. Reduced structural connectivity may also hinder this integration; indeed, ADHD patients have been shown to have anomalous white matter integrity in fronto-striatal and premotor (PM) regions relative to age matched controls (Ashtari et al., 2005; Silk et al., 2009; Konrad and Eickhoff, 2010).

#### ADHD summary

In summary, we hypothesize, with others, that motivational impairments in ADHD arise due to an inability to accurately predict the occurrence of rewarding outcomes. This in turn reduces the salience of reward predictive cues and optimal actions potentially contributing to attentional deficits. Dopamine dysfunction within the striatum seems to be a key factor in this contingency awareness impairment. Furthermore, a greater reliance on recent rather than longer-term reinforcement history could explain the rapid extinction of learnt associations, and why patients with ADHD respond better to continuous reinforcement schedules.

### Depression

The major diagnostic guidelines state that individuals experiencing depressive episodes often have difficulty making decisions (DSM IV, APA, 2000; ICD-10, WHO, 1992). Traditionally, it has been assumed that this was due to primary motivational impairments, however cognitive deficits associated with the disorder are becomingly increasingly well documented (Lee et al., 2012). We predict that whereas outcome valuation will be strongly affected in those experiencing anhedonia, contingency sensitivity impairments may also be detected in a subset of cognitively-impaired patients. Further, reward learning and cognitive deficits may persist during periods of euthymia, predisposing individuals to future depressive episodes.

### Deficits in reward sensitivity

Depression is commonly characterized by blunted reward responsiveness (Henriques and Davidson, 2000; Pizzagalli et al., 2008; McFarland and Klein, 2009) and behavioral neglect of positive stimuli (Clark et al., 2009), which is reflected in the symptoms of anhedonia, social withdrawal and reduced activity level. As experienced rewards are no longer pleasurable, it is easy to envisage how action control could become biased away from goal-directed actions toward habits, which require only the preservation of a sufficient reinforcement signal to form stimulus-response associations.

During both reward and punished responding in depressed subjects, blunted responses are observed in the medial caudate and ventromedial OFC (Elliott et al., 1998). This supports behavioral accounts of blunted reward sensitivity. Interestingly, McCabe et al. (2009) found that, in remitted depressed patients, there were decreased reward responses in the ventral striatum, caudate and anterior cingulate, despite subjective ratings being the same as controls, suggesting that altered reward sensitivity occurs independent of mood symptoms, and may actually be a predisposing factor in the etiology of depressive episodes.

One prominent theory proposes that a defect in the top-down inhibition of the amygdala by the vmPFC may underlie depression symptoms (Myers-Schulz and Koenigs, 2011). For instance, Friedel et al. (2009) reported a negative correlation between depressive symptom severity and connectivity between the mOFC and the amygdala. As discussed earlier, the amygdala and OFC and their connectivity are required for the encoding and use of value-based information. Therefore impairment in either region, or reduced connectivity between them, will likely hamper the updating of value and its integration to mediate goal-directed choice. Due to reduced OFC-BLA connectivity, we predict that individuals with severe anhedonia will be unable to alter their choices appropriately after outcome devaluation.

Significantly reduced ventral striatal activity to positive stimuli has also been observed in depressed patients (Epstein et al., 2006; Robinson et al., 2012; Stoy et al., 2012), which may reflect a deficit in using value information to guide action selection. These studies employed predominantly Pavlovian learning processes and, therefore, the focus was generally on assessing anticipation of reward rather than how value knowledge was used to guide instrumental choices. Nevertheless, Stoy et al. (2012) discovered that treatment with the common antidepressant, escitalopram, normalized anticipatory reward signals in the ventral striatum, highlighting how medications affecting reward circuitry could be effective in improving depressive symptoms. In addition, deep brain stimulation to the bilateral NAc in refractory depression has shown promising results for reduction of the symptoms of anhedonia (Schlaepfer et al., 2008; Malone et al., 2009).

### Deficits in contingency awareness

Although it is evident that anhedonia diminishes the impact of reward processes in goal-directed action, there is significantly more debate about how causal awareness is affected in depression. Depressed individuals often experience symptoms of learned helplessness, which may reflect dysfunction in causal knowledge. Learned helplessness is essentially an error in attribution of control (Miller and Seligman, 1975) in the sense that a depressed person may have aberrant beliefs about the causality of their actions in achieving a goal, or the lack thereof, and so not initiate an action. Using Bayesian modeling, Lieder et al. (2013) argued that generalization of action-outcome contingencies is able to account for a range of learned helplessness phenomena. By this account, individuals attribute outcomes to their current situation or state rather than to the chosen action; they generalize across available actions, with the belief that the state will determine the outcome, irrespective of their actions.

Paradoxically however, a large body of research has also supported the idea that dysphoric or depressive individuals often have *greater* causal sensitivity, an effect referred to as depressive realism (Alloy and Abramson, 1979; Martin et al., 1984; Benassi and Mahler, 1985; Ackermann and DeRubeis, 1991; Allan et al., 2007; Msetfi et al., 2012). Indeed, Alloy and Abramson (1979) found that, during a task incorporating both contingent and non-contingent outcomes non-depressed people were more likely to believe that their actions were causal of the outcome whereas depressed people did not show this illusion of control, and tended to rate their actions in this task as less causal.

These contradictory findings in depressed people might be reconciled by considering the role of competition between actions and cues for causal learning. There are two major predictors of outcomes in our environment: our own instrumental actions and situational stimuli such as Pavlovian cues. These two classes of events will compete as causes for outcomes of interest during causal learning tasks, like those described above. In such tasks, when non-contingent outcomes are provided, situational stimuli can become better predictors of those outcomes than actions. So the illusion of control could reflect a disposition to assign causal status to ones own actions over situational stimuli, even when situational stimuli are better predictors. In contrast, if action-outcome contingency awareness is impaired, then situational stimuli should be predicted to outcompete actions for association with specific outcomes and in their attribution as causes of those outcomes. This should be anticipated to produce more accurate causal judgments of actions, consistent with depressive realism. Furthermore, the deficit in action-outcome contingency awareness will still produce learned helplessness.

An implication of this argument, derived from the distinct neural regions responsible for action-outcome vs. stimulus-outcome contingency awareness, is that pathology in depression should be restricted to those medial prefrontal cortical regions that are critical for A-O learning. Conversely, the lateral PFC regions implicated in S-O learning should be relatively intact on this view. In fact, considerable research has explored the role of mPFC in behavioral control over the effects of chronic stress (Amat et al., 2005; Maier and Watkins, 2010). Resistance to environmental stressors, and as such, resilience against feelings of helplessness, is thought to rely on inhibitory control exerted by the vmPFC over limbic structures. Without this inhibition, it is argued, stressors could cause sensitization of serotonergic neurons in the dorsal raphe, changing how the organism responds to subsequent aversive stimuli (Maier and Watkins, 2005).

Serotonin is a neuromodulator thought to play a key role in the neurochemical basis of depression, with selective serotonin reuptake inhibitors being a first-line treatment of depression. It has also been implicated in the modulation of decision processes. For instance, Doya (2002) proposed that low levels of serotonin may be associated with excessive discounting of future rewards, while others have argued that it is more specifically involved with inhibiting actions and thoughts associated with aversive outcomes (Daw et al., 2002; Dayan and Huys, 2008; Huys et al., 2012; Robinson et al., 2012). This view proposes that serotonin reductions enhance punishment predictions, but do not effect reward predictions. This raises another interesting line of research–whether individuals with depression are perhaps better at learning associations with negative rather than positive consequences (see Eshel and Roiser, 2010, for a review). Numerous studies have demonstrated that depressed individuals exhibit hypersensitivity to negative feedback (Elliott et al., 1997), and hyposensitivity to positive feedback (Pizzagalli et al., 2008), and highlight how aberrance in evaluation, and subsequent allocation of attention, has detrimental effects on contingency learning.

The emerging field of computational psychiatry has provided a promising new avenue for understanding psychiatric illnesses, through applying mathematical models to behavioral and biological problems. Within decision neuroscience, it aims to provide a systematic explanation of the core processes in decision-making in a manner consistent with neurobiologically relevant processes (Dayan and Huys, 2008). A series of studies have recently used this approach in discerning the specific decision-making deficits at play in depression. In this approach, reward sensitivity is related to valuation, while learning rate represents a dimension of contingency awareness. Chase et al. (2010) found a reduced learning rate in depression, however they did note that learning rate was more closely related to severity of anhedonia than diagnosis *per se*. A recent meta-analysis in un-medicated depression reported that reduced reward sensitivity (reduced prediction errors) had greater affect than learning rate on overall learning performance, and was correlated with anhedonia severity (Huys et al., 2013). This is supported by reduced striatal activation during reward receipt (Pizzagalli et al., 2009; Smoski et al., 2009). Using a medicated sample, however, we found that learning rate was reduced in depression, which may indicate that while overall choice behavior remains impaired, antidepressant medication may change the dynamics of the contributing processes (Griffiths et al., unpublished data).

### Structural and resting-state abnormalities in goal-directed circuitry

The difficulties depressed individuals have with learning and performance of goal-directed action correspond with abnormalities in learning and choice related brain regions. Gray matter volumetric studies and postmortem examinations have show neuronal size reductions relative to controls in the OFC (Cotter et al., 2005; Drevets and Price, 2008), left ACC (Drevets et al., 1997; Coryell et al., 2005), dlPFC (Drevets, 2004), caudate and NAc (Baumann et al., 1999). Moreover, symptoms of anhedonia, depression severity and probability of suicide have all been associated with reduced caudate volume (Pizzagalli et al., 2008) and caudate activity (Forbes et al., 2009).

There is a complex relationship between depression severity and the OFC. Some studies report increased OFC activity in treatment responsive depressives, whereas more severely ill patients have relatively normal or decreased OFC metabolism (Drevets et al., 1997; Mayberg, 1997). Drevets et al. (1997) posit that increased OFC activity may reflect a cognitive compensatory effort to attenuate negative emotion, while reduced OFC activity may reflect a primary pathology related to monoamine dysfunction. This is supported by enhanced dextroamphetamine-induced rewarding effects compared to controls (Tremblay et al., 2002, 2005). Functional imaging during a range of tasks involving planning, reward, behavioral choice and feedback have reported abnormal recruitment of the mOFC (Elliott et al., 1998; Taylor Tavares et al., 2008), and lesions of the human OFC have been argued to increase the risk for developing depression (Drevets, 2007), although this is controversial (see e.g., Carson et al., 2000). Nevertheless, reports that this region plays a key role in valuation suggest that any compromised function will likely affect goal-directed action.

In addition to problems with the core circuitry associate with goal-directed action, imaging studies have shown abnormally low dlPFC activity during resting state (Galynker et al., 1998), yet overly activated activation during working memory and cognitive control tasks (Harvey et al., 2005; Wagner et al., 2006), potentially indicating inefficiency in this cognitive control region. This may contribute to the increased indecisiveness experienced in depression.

### Depression summary

In summary, depression is characterized by impairments in reinforcement learning, and using affective information to guide behavior. Anhedonia, a common symptom in depression, maps closely onto deficits within outcome valuation circuitry, and is the clearest example of how problems with reward value lead to reductions in goal-directed action. Learned helplessness, or a lack of resistance to environmental stressors, may also occur when S-O associations outcompete A-O associations. This may cause depressed individuals to generalize action-outcome contingencies across different contexts, and become less adaptive to new environments.

## Other disorders

It is clear that an associative learning framework can provide testable hypotheses and explanations for a range of deficits in clinical disorders. Though we can only provide a brief discussion of three such disorders here, the potential exists for many others. For instance, Obsessive-Compulsive disorder, where behavior may exhibit an overreliance on habits due to dysfunctional goal-directed circuitry (Gillan et al., 2011), and anorexia nervosa, where there is a tendency to deprive oneself of food, despite, or likely because of, hyperactivity in evaluative neural circuitry during food presentation (Keating et al., 2012), provide interesting examples.

Importantly, assessment of decision-making deficits need not be constrained rigidly by diagnostic classifications. Most psychiatry research uses these classifications with the assumption that it will provide a homogenous subset of participants. However multiple systems may be differentially affected in these patients, and comorbidities and group averaging may contaminate both behavioral and neural results. Further, symptom commonalities also occur across diagnostic boundaries, for instance anhedonia, which can occur in a range of disorders, such as depression, post-traumatic stress disorder and schizophrenia. Thus, behavioral tests that probe specific processes and neural deficits could have great value in guiding research on biologically-based individualized classification.

It is worth mentioning that the wide-ranging use of medications and substance use in psychiatric groups makes testing these populations to clearly delineating the source of their illness very challenging. Most medications affect multiple, predominantly monoamine, neurotransmitter systems, and variance in functional effects occurs over different doses. These neurotransmitter systems are intricately involved in reward and decision processes, thus it can be difficult to distinguish disorder-related findings from those induced by medication, and to untangle the differential effects of medications across tasks. For instance, using SPECT, Paquet et al. (2004) found a correlation between procedural learning ability and D2 receptor occupancy. Patients on second generation antipsychotics (SGA) perform better at procedural learning tasks compared to those on first generation antipsychotics (FGA), which is thought to be due to the comparatively lower affinity for striatal D2 receptors in SGAs (Stevens et al., 2002; Scherer et al., 2004). Conversely, Beninger et al. (2003) found that SGAs adversely affected performance on the IGT, which they surmise may be due to the high affinity of SGAs for serotonin receptors in the PFC.

## Conclusions

Though much progress has been made in elucidating the processes and neurobiology of decision-making, a great deal remains to be done. Contradictory findings and interpretations persist, and with contributions from diverse fields such as economics, computer science and psychology, a “common language” has not yet been achieved. Decision-making is an extremely complex process, and as such, the range of tasks used to assess this skill is broad. Great care must be taken when comparing results across tasks, as task-related variables may modulate the underlying circuitry involved.

A key strength of associative learning tasks is the strong theoretical basis, and the broad foundation of animal research that has helped develop our knowledge of the circuitry underlying specific learning processes. By establishing links between well-defined psychological processes (e.g., goal-directed action), neural circuits and even intracellular signaling, we can develop a biologically-based phenotype of psychopathology, grounded in translatable behavioral tests. Nevertheless, important questions remain regarding how we conceptualize the interaction between these learning systems. For instance, a flat architecture assumes that goal-directed and habitual processes exist in parallel, with an arbitrator determining which system is utilized for the following action. A hierarchical structure, however, proposes a global goal-directed system that incorporates habitual action sequences when they can achieve the desired goal. Although beyond the scope of this review, there are a number of neural and computational theories that debate how and where action values are compared and transformed into motor signals, and if in fact, cognitive action selection and motor planning occur as serial or simultaneous processes (Cisek and Kalaska, 2010; Hare et al., 2011; Cisek, 2012; Rushworth et al., 2012; Wunderlich et al., 2012; Dezfouli and Balleine, 2013). These theories are important considerations for determining precisely how fundamental processes such as outcome valuation and contingency learning are transformed into the motor choices producing goal-directed performance.

Decision neuroscience is an exciting field that incorporates translational research from a range of species and scientific techniques. Within this field, associative learning accounts have provided a theoretical basis for the development of a range of biologically relevant behavioral paradigms. This framework endeavors to draws together behavioral and neurological processes, creating impetus for a wide range of testable hypotheses. Through systematic application of biologically relevant paradigms, we could further identify specific problems contributing to maladaptive decision-making across psychiatric disorders. This review has attempted to highlight how a number of deficits across psychiatric disorders may be explained in terms of fundamental reward learning and performance impairments, which could shed some new light on the functional impairment and neurobiological underpinnings of these illnesses.

## Conflict of interest statement

The authors declare that the research was conducted in the absence of any commercial or financial relationships that could be construed as a potential conflict of interest.
